# Protein and Immune Component Content of Donor Human Milk in Japan: Variation with Gestational and Postpartum Age

**DOI:** 10.3390/nu15102278

**Published:** 2023-05-11

**Authors:** Miori Tanaka, Midori Date, Kumiko Miura, Mizuho Ito, Noriko Mizuno, Katsumi Mizuno

**Affiliations:** 1 The Nippon Foundation Human Milk Bank, 17-10 Nihonbashi-koamicho, Chuo-ku, Tokyo 103-0016, Japan; midori.date@milkbank.or.jp (M.D.); kumiko.urakami@gmail.com (K.M.); 11119011m@stud.showa-u.ac.jp (M.I.); katsuorobi@med.showa-u.ac.jp (K.M.); 2 Faculty of Medicine, Oita University, 1-1 Hasamamachiidaigaoka, Yufu-shi, Oita 879-5593, Japan; 3 School of Medicine, Showa University, 1-5-8 Hatanodai, Shinagawa-ku, Tokyo 142-8555, Japan; 4 Japan Human Milk Bank Association, 4-4 Nihonbashi-hisamatsucho, Chuo-ku, Tokyo 103-8480, Japan; norarobi3@gmail.com; 5 Department of Pediatrics, School of Medicine, Showa University, 1-5-8 Hatanodai, Shinagawa-ku, Tokyo 142-8555, Japan

**Keywords:** donor human milk, human milk bank, protein, sIgA, lactoferrin, gestational age, postpartum week

## Abstract

Donor human milk (DHM) is the second-best nutrition for preterm infants when their own mother’s milk is unavailable. The nutrient content of human milk is influenced by various factors, including gestational and postpartum age, but there are no data regarding DHM composition in Japan. The aim of this study was to determine the protein and immune component content of DHM in Japan and to elucidate the effects of gestational and postpartum age on nutrient composition. From September 2021 to May 2022, 134 DHM samples were collected from 92 mothers of preterm and term infants. Protein concentrations in preterm DHM (*n* = 41) and term DHM (*n* = 93) were analyzed using a Miris Human Milk Analyzer. The concentrations of secretory immunoglobulin A (sIgA) and lactoferrin, major immune components, were measured using enzyme-linked immunosorbent assays. Preterm DHM exhibited higher protein content than term DHM (1.2 g/dL and 1.0 g/dL, respectively, *p* < 0.001), whereas sIgA content was higher in term DHM than in preterm DHM (110 μg/mL and 68.4 μg/mL, respectively, *p* < 0.001). Gestational age was negatively correlated with protein levels and positively correlated with sIgA and lactoferrin levels. Furthermore, a negative correlation was found between postpartum week and protein, sIgA, and lactoferrin concentrations. Our data suggest that gestational and postpartum age affects protein, sIgA, and lactoferrin concentrations in DHM. These results indicate the importance of nutritional analysis for the appropriate use of DHM in preterm infants.

## 1. Introduction

Human milk is a unique source of essential nutrients and is regarded as the gold standard for protective nutrients fed to all preterm and term infants. Exclusive breastfeeding for 6 months is recommended by the World Health Organization and national institutions in many countries [1,2,3]. Epidemiological studies have shown that breastfed infants are better protected than formula-fed infants against necrotizing enterocolitis (NEC), sepsis, and mortality, especially in preterm infants [4,5,6]. In addition to macronutrients and energy necessary for growth, human milk contains various bioactive components, including immune proteins, growth factors, metabolic hormones, and oligosaccharides, which contribute to immune maturation and organ development [7,8]. Immune proteins, such as secretory immunoglobulin A (sIgA) and lactoferrin, are more abundant in human milk than in animal milks and formula [9,10] and play an important role in preventing NEC or late-onset sepsis in preterm infants [11,12,13].

Donor human milk (DHM) is widely recommended for preterm and/or very low birth weight infants when their own mother’s milk is unavailable [14,15]. According to previous trials, the most important reason for providing DHM instead of formula for preterm infants is the reduction of NEC [16]. DHM also allows mothers time to establish their milk supply with the provision of lactation support. In human milk banks, breast milk from donors is pasteurized, tested for bacteria, and frozen for storage. Upon request from neonatal intensive care units (NICUs), human milk banks provide DHM for infants born preterm and/or with very low birth weight. The number of human milk banks is increasing worldwide, with more than 750 banks operating in over 60 countries [14]. In Japan, the first public human milk bank, the Japan Human Milk Bank Association (JHMBA), was opened in 2017. The Nippon Foundation Human Milk Bank (TNFHMB) was established in 2021 as the second human milk bank in Japan. Currently, there are only 2 human milk banks in Japan, TNFHMB, and JHMBA, and the number of donors and recipients in 2022 was approximately 600 and 800, respectively. In addition to supplying DHM, TNFHMB conducts research on human milk, including the evaluation of DHM composition.

The composition of human milk is highly variable and is influenced by numerous factors, including maternal factors such as health, age, and diet; infant factors such as gestational age, gender, and birth weight; and physiological factors such as lactation stage (colostrum, transitional, and mature), length of feed (foremilk or hindmilk), and circadian rhythm [7,17]. In particular, the relationship between gestational and postpartum age and the macronutrient and immune component content of human milk has been widely investigated [18,19,20]. Changes in human milk composition are essential for infant health and growth, corresponding to individual infant requirements. Preterm infants have higher protein and bioactive component requirements than term infants, and nutrient variability in human milk may contribute to growth deficits in preterm infants [21]. In human milk banks, there are several types of DHM, including preterm and term DHM, with varying nutritional components. Therefore, a concern about using DHM for premature infants is that fortification may be required to meet preterm nutritional requirements. Additionally, previous studies have revealed that the pasteurization process greatly reduces bioactive components in human milk [22].

The nutrient content of human milk is influenced by various factors and related to infant growth; however, there are no studies that have evaluated DHM composition in Japan. The aim of this study was therefore to determine the protein and immune component content of DHM in Japan and to clarify what kind of donors should be selected to provide appropriate DHM to preterm infants.

## 2. Materials and Methods

### 2.1. Study Design

This was a cross-sectional study to determine the protein and immune component contents of DHM in Japan. This study was conducted at TNFHMB between September 2021 and May 2022. The study was conducted according to the guidelines of the Declaration of Helsinki, and ethical approval was obtained from Showa University Research Ethics Review Board (approval number: 2714). All donors provided written consent for using their human milk for research purposes.

### 2.2. Milk Samples

A total of 134 DHM samples were collected from 92 mothers, 26 with preterm (gestational age < 37 weeks) and 66 with term (gestational age ≥ 37 weeks) infants. In this study, donors were limited to those with Japanese nationality. Because of the homogeneity of the Japanese population, it can be reasonably assumed that the donors were nearly all of Japanese ethnicity. All donors had completed a screening process consisting of a health questionnaire and a medical interview with doctors or midwives who reviewed the health questions and assessed the donor’s suitability. Blood screening test for the human immunodeficiency virus (HIV I/II), the human T-cell leukemia virus (HTLV I/II), hepatitis B and C, and syphilis was then undertaken. The donors were screened based on their detailed medical history, drug history, physical examinations, and laboratory data, and donors who did not meet the eligibility criteria were excluded. Milk was obtained by hand or pump expression, and each donor was taught the collecting techniques by our midwives. The expression equipment was provided free of charge by TNFHMB or JHMBA upon the donor’s request. After expression, milk in clean bags was frozen and stored in a freezer at the donor’s home. Milk was then sent to TNFHMB or JHMBA through a shipping company contracted by TNFHMB and JHMBA under conditions of −15 °C (or lower). On arrival at TNFHMB or JHMBA, all milk was checked to ensure that it remained frozen and that there was no damage to the sterile bags or the presence of foreign matter. Subsequently, milk was stored at −30 °C until pasteurization. Frozen milk was thawed in a refrigerator overnight and then pasteurized within 24 h. In preparation for pasteurization, thawed milk was pooled in a batch with other milk from the same donor to reduce nutrient variability that may occur under a variety of conditions, including time of day and whether expression occurs before or after breastfeeding. Holder pasteurization (62.5 °C, 30 min) was performed with a Sterifeed S90 pasteurizer (MediCare Colgate Ltd., Kentisbeare, UK), Barkey clinitherm pasteur 10/80 (Barkey GmbH & Co., KG, Leopoldshöhe, Germany), or Racoon dry pasteurizer HMP-4 (Mita Rika Kogyo Co., Ltd., Osaka, Japan). DHM samples were taken from each milk batch after pasteurization and stored at −30 °C to measure protein levels and at −80 °C to measure immune component levels until analysis. The dates of the first and last milk expression and pasteurization are routinely recorded in the database. DHM samples were used for research if the period between the first and last expression dates was less than 1 month. If this period overlapped from one postpartum week to the next, the DHM postpartum week number was assigned as the week number of the first expression date.

### 2.3. Measurement of Proteins in DHM

Protein content was measured via mid-infrared transmission spectroscopy using a Miris Human Milk Analyzer (Miris AB, Uppsala, Sweden) according to the manufacturer’s instructions. DHM samples were heated to 40 °C in a thermal block and homogenized at 1.5 s/mL using a Miris Ultrasonic Processor (Miris AB), and then 2.5 mL of sample was injected into the cuvette via syringe. A daily quality control, as well as calibration, checks, and cleaning steps, was performed using a Miris Calibration Control Kit™, Miris Check™, and Miris Cleaner™ (Miris AB) prior to the analysis of the DHM samples. The analyzer has specific waveband filters that are selected to absorb only mid-infrared radiation correlated to each macronutrient in human milk. The waveband used for crude and true protein determination is 6.5 μm and assesses the amount of radiation absorbed by secondary amide groups of peptide bonds, specific functional groups of the protein. The quantitative determination of protein is performed according to the Beer–Lambert law, and values are presented as g/dL of milk. The internal calibration is based on chemical standard methods validated for analysis of human milk: Kjeldahl (nitrogen × 6.38) for crude protein (ISO 8968-1). Crude protein represents the protein values in this study. The analyzer has demonstrated repeatability (SD) of ≤0.05 g/100 mL and accuracy of ±15% for protein, and has been previously shown to be adequate for macronutrient measurement of human milk [23].

### 2.4. Measurement of Immune Components in DHM

DHM samples were centrifuged at 3000× *g* for 15 min at 4 °C, separating the fat layer and enabling collection of the lower aqueous layer for analysis. Enzyme-linked immunosorbent assay (ELISA) was used to quantify the levels of sIgA and lactoferrin according to the manufacturer’s instructions. The concentrations of sIgA were measured using a Secretory IgA (Human) ELISA Kit (Abnova, Taipei City, Taiwan) with a sensitivity of 0.6 μg/mL. Lactoferrin levels were measured using a Human Lactoferrin ELISA kit (BioVendor, Brno, Czech Republic) with a sensitivity of 0.4 ng/mL.

### 2.5. Statistical Analysis

Statistical analyses were performed using the GraphPad Prism 9 software package (GraphPad Software, La Jolla, San Diego, CA, USA). All continuous variables were tested for normality by a D’Agostino–Pearson test. Maternal age, gestational age, postpartum week, infant birth weight, and concentrations of protein, sIgA, and lactoferrin are shown as the median and interquartile range (IQR) since they exhibit nonparametric distribution. Comparisons between preterm and term groups were performed using a Mann–Whitney U test. Differences in DHM composition at different postpartum weeks were assessed using a Kruskal–Wallis test followed by Steel–Dwass test. Relationships between gestational age and DHM composition and between postpartum week and DHM composition were determined using Spearman’s rank correlation coefficient. Differences were considered statistically significant when *p* < 0.05.

## 3. Results

### 3.1. Demographic Characteristics of Subjects and Samples

The demographic characteristics of the mothers and infants are compared in Table 1. Among 92 mothers, 26 mothers delivered preterm infants at <37 weeks gestation, and 66 mothers delivered term infants at ≥37 weeks gestation. A total of 134 DHM samples were collected in the study, including 41 samples collected from preterm mothers and 93 samples collected from term mothers. In some instances, a single donor provided milk multiple times during the study period. The median maternal age of mothers of preterm and term infants was 36 years and 34 years, respectively (*p* = 0.462). There were significant differences in gestational age (preterm: 29 weeks vs. term: 39 weeks; *p* < 0.001), postpartum week (preterm: 10 weeks vs. term: 24 weeks; *p* < 0.001), and infant birth weight (preterm: 1097 g vs. term: 3002 g; *p* < 0.001).

### 3.2. Protein and Immune Component Levels in Preterm and Term DHM

Protein and immune component levels in preterm and term DHM are shown in Table 2. Protein levels were significantly higher in preterm DHM than in term DHM (1.2 g/dL and 1.0 g/dL, respectively, *p* < 0.001), whereas sIgA levels were significantly higher in term DHM compared with preterm DHM (110 μg/mL and 68.4 μg/mL, respectively, *p* < 0.001). No significant differences were seen in lactoferrin levels of the preterm and term DHM (106 μg/mL and 90.3 μg/mL, respectively, *p* = 0.248).

### 3.3. Protein and Immune Component Levels in Relation to Gestational Age

**Figure 1:**
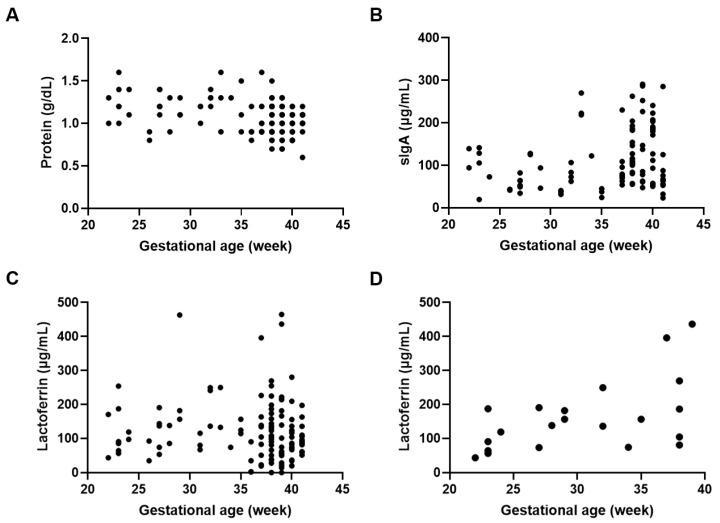
Protein and immune component concentrations in relation to gestational age. Correlation between gestational age and (**A**) protein levels in DHM (r = −0.334, *p* < 0.001), (**B**) sIgA levels in DHM (r = 0.206, *p* < 0.05), (**C**) lactoferrin levels in DHM (r = −0.103, *p* = 0.246), and (**D**) lactoferrin levels in DHM collected 0 to 8 weeks postpartum (r = 0.531, *p* < 0.05).

Various factors could affect the protein and immune component levels in DHM. We determined the effect of gestational age on protein and immune component levels and found that protein levels were negatively correlated with gestational age (*p* < 0.001; r = −0.334) when all postpartum weeks were analyzed simultaneously (Figure 1A). For immune components, simultaneous analysis of all postpartum weeks revealed a positive correlation between sIgA levels and gestational age (*p* < 0.05; r = 0.206) (Figure 1B), whereas no correlation was found between lactoferrin levels and gestational age (Figure 1C). However, a positive correlation was seen between gestational age and lactoferrin levels in DHM collected between 0 and 8 weeks postpartum (*p* < 0.05; r = 0.531) (Figure 1D).

### 3.4. Protein and Immune Component Levels in DHM at Different Postpartum Weeks

Protein and immune component levels in preterm DHM at different postpartum weeks are shown in Table 3. Protein levels in DHM at 0–8 weeks postpartum were significantly higher than at 9–16 weeks (1.3 g/dL vs. 1.2 g/dL, respectively; *p* < 0.05) and >17 weeks postpartum (1.3 g/dL vs. 0.8 g/dL, respectively; *p* < 0.001). sIgA levels were comparable between the groups, whereas lactoferrin levels in DHM at 0–8 weeks postpartum tended to be higher than >17 weeks postpartum (138 µg/mL vs. 85.1 µg/mL, respectively; *p* = 0.060).

Protein and immune component levels in term DHM at different postpartum weeks are shown in Table 4. Protein levels in DHM at 0–8 weeks postpartum were significantly higher than at 9–16 weeks (1.3 g/dL vs. 1.1 g/dL, respectively; *p* < 0.01) and >17 weeks postpartum (1.3 g/dL vs. 1.0 g/dL, respectively; *p* < 0.001). Although there were no significant differences in sIgA levels, lactoferrin levels in DHM at 0–8 weeks postpartum were significantly higher than at >17 weeks postpartum (270 µg/mL vs. 85.8 µg/mL, respectively; *p* < 0.05).

### 3.5. Protein and Immune Component Levels in Relation to Postpartum Week

**Figure 2:**
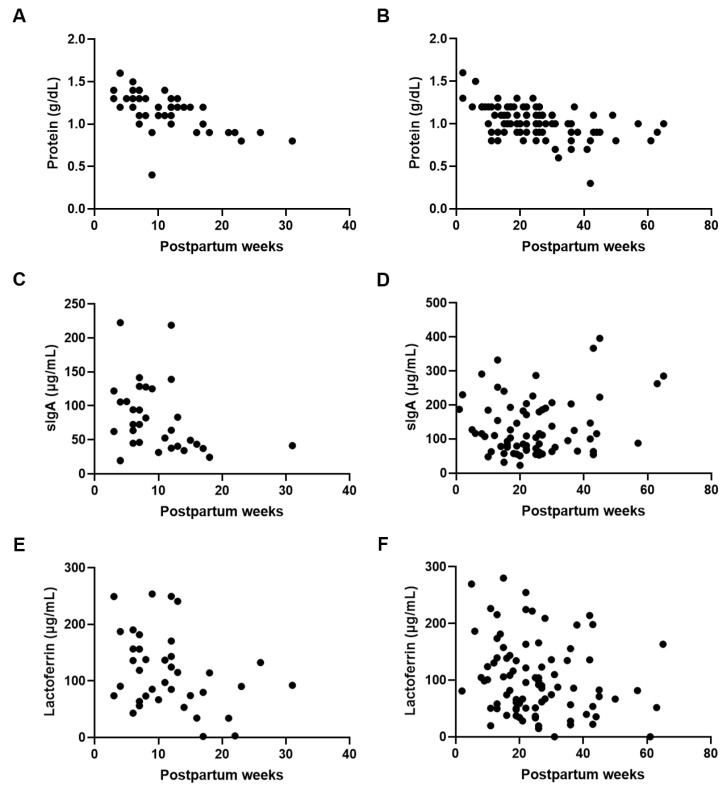
Protein and immune component concentrations in relation to postpartum week. Correlation between postpartum week and (**A**) protein levels in preterm DHM (r = −0.672, *p* < 0.001), (**B**) protein levels in term DHM (r = −0.495, *p* < 0.001), (**C**) sIgA levels in preterm DHM (r = −0.400, *p* < 0.05), (**D**) sIgA levels in term DHM (r = 0.055, *p* = 0.644), (**E**) lactoferrin levels in preterm DHM (r = −0.322, *p* < 0.05), and (**F**) lactoferrin levels in term DHM (r = −0.200, *p* = 0.063).

Next, we confirmed the effect of postpartum week on the composition of the preterm and term DHM. Protein levels were negatively correlated with postpartum week in both preterm (*p* < 0.001; r = −0.672) and term (*p* < 0.001; r = −0.495) DHM (Figure 2A,B). A negative correlation between sIgA concentrations and the postpartum week was only seen for preterm DHM (*p* < 0.05; r = −0.400) (Figure 2C,D). Lactoferrin levels were negatively correlated or tended to be correlated with postpartum week in both preterm (*p* < 0.05; r = −0.322) and term (*p* = 0.063; r = −0.200) DHM (Figure 2E,F).

## 4. Discussion

This was the first study in Japan to determine the protein and immune component content of DHM. We found that preterm DHM had higher protein content than term DHM, whereas sIgA content was higher in term DHM. The median protein content was similar to that of previous studies [20,24], which reported preterm DHM protein concentrations of 1.0–1.1 g/dL and term DHM protein concentration of 0.8 g/dL. These studies reported a difference of approximately 0.2 g/dL between DHM protein levels of preterm and term donors, which is consistent with our data. Although we expected preterm DHM to be valuable due to high protein levels, it is likely that term DHM is also important given the high sIgA levels.

Protein in human milk is a major determinant of physical growth, and previous evidence has shown that greater protein intake was associated with higher body length in preterm infants [21,25]. Additionally, protein intake during the first or second week of life was associated with the mental developmental index at 18 months [25] and total brain volume at term-equivalent age [26] in preterm infants. Thus, nutritional status after birth is directly related to both future growth and cognitive function outcomes of preterm infants. In the present study, an inverse relationship between protein concentrations and gestational age was observed, which corresponds to previous reports [18,20,27]. This may be attributed to increased levels of total protein in preterm milk associated with incomplete differentiation of mammary epithelial cells, reduced blood flow, absence of tight junctions between epithelial cells, and maternal hypertension [28,29]. Regarding postpartum week, previous findings showed the highest protein content in the first week of lactation with a gradual decline over subsequent weeks [18,30,31]. This is consistent with our results that DHM protein composition changed over time, with an inverse relationship between protein content and postpartum week for both preterm and term DHM. Preterm donors are more likely to express breast milk while their infants are hospitalized in NICUs, and were therefore in earlier postpartum weeks compared with the term donors in this study, which may contribute to the higher protein concentrations.

Immune components in human milk, such as sIgA and lactoferrin, enhance the neonatal immune system and reduce preterm birth complications. sIgA is the most abundant antibody in human milk and provides critical neonatal intestinal defense by preventing the entry of pathogens [32]. Lactoferrin is an iron-binding glycoprotein that exhibits antibacterial, antiviral, and anti-inflammatory functions and enhances host defense by sequestering iron necessary for bacterial growth [33]. It was shown that a relative decrease in IgA-bound bacteria was associated with NEC development in preterm infants [13], whereas oral administration of IgA-IgG prevented NEC in low birth weight infants when breast milk from their mothers was not available [34]. A recent systematic review and meta-analysis that included nine randomized controlled trials reported that prophylactic lactoferrin significantly reduced the incidence of NEC and late-onset sepsis in preterm infants [35]. Although previous studies reported no significant differences in sIgA and lactoferrin concentrations for different gestational age groups [36,37], we found that sIgA and lactoferrin levels in DHM were positively correlated with gestational age at all postpartum weeks and 0 to 8 weeks postpartum, respectively. A positive correlation between sIgA and gestational age was also observed at 0 to 8 weeks postpartum (data not shown). It was previously reported that the mean concentrations of sIgA and lactoferrin are inversely related to the volume of milk expressed [36,38], and that expressed milk volume is higher for preterm mothers compared with term mothers [36]. The increase in sIgA and lactoferrin levels with gestational age may therefore be due to differences in milk volume between the preterm and term donors. However, since mothers of preterm infants are generally unable to express sufficient amounts of milk [39], further research is needed to elucidate the reasons for the discrepancy among previous findings and our own.

In this study, sIgA and lactoferrin levels decreased as the number of postpartum weeks increased, which was in accordance with previous studies [36,37,40,41]. High sIgA and lactoferrin concentrations at an early stage of lactation may be associated with neonatal immune development and the beneficial effects of human milk. A previous in vitro study has demonstrated that lactoferrin and its peptides stimulated intestinal proliferation at high concentrations, while low concentrations of lactoferrin promoted intestinal differentiation [42]. Thus, the changes in sIgA and lactoferrin concentrations seen in this study may reflect the concentration-dependent biological functions of immune components during different stages of infant growth. Combined with the results that protein levels decreased over time, DHM with earlier postpartum weeks tended to have higher protein, sIgA, and lactoferrin levels, regardless of preterm or term delivery. It should be noted that DHM with a large number of postpartum weeks may have lower protein content and some DHM may not meet the nutritional requirements of preterm infants with standard fortification.

Nutrient variability in human milk may result in poor postnatal growth of preterm infants [21], and nutritional analysis of DHM would assist in assigning the most appropriate DHM to each preterm infant, such as assigning DHM with higher protein and immune component levels to preterm infants with shorter gestational age and lower weight. Furthermore, individual fortification, including adjustable fortification and targeted fortification, is encouraged to optimize nutrient intake [43], and previous reports have indicated that individual fortification after nutritional analysis improved the weight and growth of preterm infants compared with standard fortification [44,45]. Individual fortification is time-consuming and expensive, but measuring the nutrient composition of DHM may expand its use in NICUs. Although none of the DHM in this study had extremely low protein levels compared with non-pasteurized human milk, some DHM had low sIgA and lactoferrin levels after pasteurization. Heat treatments such as Holder pasteurization significantly decrease the concentration of immunological components, such as sIgA and lactoferrin, in human milk [22]. Pooling DHM from multiple donors based on nutritional analysis has been shown to reduce variability in protein, fat, and IgA content [46,47]. In Japanese human milk banks, as well as several overseas banks, both DHM from a single donor and DHM from multiple donors are provided. Previous findings and our data indicate the need for nutritional analysis of DHM, especially for preterm infants with poor postnatal growth.

There are several limitations to this study. First, the number of preterm (*n* = 26) and term (*n* = 66) donors was not balanced, and the number of preterm donors was small. This may be due to the fact that TNFHMB was established in 2021, and the human milk bank system in Japan is still underdeveloped compared with that in other countries. However, this is the first study in Japan to report the protein, sIgA, and lactoferrin content of DHM and the effects of gestational and postpartum age on nutrient composition. The present results therefore may not be applicable to other populations. Second, we did not evaluate fat and carbohydrate levels in DHM. Fat content in human milk is higher in hindmilk than in foremilk [48]. In this study, DHM was used as the sample and it was therefore not possible to distinguish between foremilk and hindmilk. Furthermore, carbohydrate was the least variable macronutrient in previous studies [20]. For these reasons, fat and carbohydrate concentrations were not evaluated in this study. Third, this was a cross-sectional study, and we did not collect information about several factors reported to influence protein, sIgA, and lactoferrin levels in human milk, including maternal diet, body mass index, circadian rhythm, methods of milk expression, and the volume of milk expressed [36,38,49,50,51,52]. It will be necessary to investigate and standardize the effects of these factors on DHM composition in the future.

## 5. Conclusions

In conclusion, we demonstrated that protein and immune component concentrations in DHM donated by Japanese women were affected by gestational and postpartum age. Overall, our results corresponded with previous foreign reports, indicating that protein, sIgA, and lactoferrin levels decrease as the number of postpartum weeks progressed. Protein levels also decreased as gestational age progressed. However, we observed an increase in sIgA and lactoferrin levels with gestational age, which is inconsistent with previous reports. These findings indicate the importance of nutritional analysis to provide DHM appropriate to the background of each preterm infant. Further research with an increased sample size is needed and the effects of confounding factors require further investigation.

## Figures and Tables

**Table 1:** Demographic characteristics of mothers and infants.

	Preterm		Term		*p* Value
	*n*	Median (IQR)	*n*	Median (IQR)	
Number of mothers	26		66		
Number of samples	41		93		
Number of samples in each postpartum category					
0–8 weeks	17		7		
9–16 weeks	16		19		
>17 weeks	8		67		
Maternal age (year)		36 (31–38)		34 (30–37)	0.462
Gestational age (week)		29 (27–34)		39 (38–40)	<0.001
Postpartum week		10 (7–15)		24 (16–32)	<0.001
Birth weight (g)		1097 (659–2006)		3002 (2763–3220)	<0.001

**Table 2:** Protein and immune component levels in preterm and term DHM.

	Preterm (*n* = 41)	Term (*n* = 93)	*p* Value
Protein (g/dL)	1.2 (1.0–1.3)	1.0 (0.9–1.2)	<0.001
sIgA (μg/mL)	68.4 (41.9–118)	110 (67.8–187)	<0.001
Lactoferrin (μg/mL)	106 (71.7–156)	90.3 (51.1–139)	0.248

Data are represented as median (IQR).

**Table 3:** Protein and immune component levels in preterm DHM at different postpartum weeks.

	0–8 Weeks (*n* = 17)	9–16 Weeks (*n* = 16)	>17 Weeks (*n* = 8)
Protein (g/dL)	1.3 (1.2–1.4) ^a^	1.2 (1.0–1.2) ^b^	0.8 (0.9–1.0) ^c^
sIgA (μg/mL)	93.8 (62.9–125)	50.9 (38.4–115)	39.3 (27.5–213)
Lactoferrin (μg/mL)	138 (73.7–189)	120 (76.8–223)	85.1 (10.7–109)

Data are represented as median (IQR). Different letters show significant differences (*p* < 0.05).

**Table 4:** Protein and immune component levels in term DHM at different postpartum weeks.

	0–8 Weeks (*n* = 7)	9–16 Weeks (*n* = 19)	>17 Weeks (*n* = 67)
Protein (g/dL)	1.3 (1.2–1.6) ^a^	1.1 (0.9–1.2) ^b^	1.0 (0.9–1.1) ^bc^
sIgA (μg/mL)	157 (116–245)	93.1 (62.9–185)	107 (65.0–189)
Lactoferrin (μg/mL)	270 (105–437) ^a^	124 (58.2–174) ^ab^	85.8 (50.5–136) ^b^

Data are represented as median (IQR). Different letters show significant differences (*p* < 0.05).

## Data Availability

The datasets used and/or analyzed during the current study are available from the corresponding author on reasonable request.
